# Comprehensive Study of Tumor Immune Microenvironment and Relevant Genes in Hepatocellular Carcinoma Identifies Potential Prognostic Significance

**DOI:** 10.3389/fonc.2020.554165

**Published:** 2020-09-24

**Authors:** Wenbiao Chen, Xujun Zhang, Kefan Bi, Hetong Zhou, Jia Xu, Yong Dai, Hongyan Diao

**Affiliations:** ^1^State Key Laboratory for Diagnosis and Treatment of Infectious Diseases, National Clinical Research Center for Infectious Disease, Collaborative Innovation Center for Diagnosis and Treatment of Infectious Diseases, School of Medicine, The First Affiliated Hospital, Zhejiang University, Hangzhou, China; ^2^Department of Clinical Medical Research Center, The Second Clinical Medical College of Jinan University, The First Affiliated Hospital Southern University of Science and Technology, Shenzhen People's Hospital, Shenzhen, China

**Keywords:** tumor immune microenvironment, gene, prognostic signature, immune activation, hepatocellular carcinoma

## Abstract

**Background:** The tumor immune microenvironment (TIME) is an external immune system that regulates tumorigenesis. However, cellular interactions involving the TIME in hepatocellular carcinoma (HCC) are poorly characterized.

**Methods:** In this study, we used multidimensional bioinformatic methods to comprehensively analyze cellular TIME characteristics in 735 HCC patients. Additionally, we explored associations involving TIME molecular subtypes and gene types and clinicopathological features to construct a prognostic signature.

**Results:** Based on their characteristics, we classified TIME and gene signatures into three phenotypes (TIME T1–3) and two gene clusters (Gene G1–2), respectively. Further analysis revealed that Gene G1 was associated with immune activation and surveillance and included CD8^+^ T cells, natural killer cell activation, and activated CD4^+^ memory T cells. In contrast, Gene G2 was characterized by increased M0 macrophage and regulatory T cell levels. After calculation of principal component algorithms, a TIME score (TS) model, including 78 differentially expressed genes, was constructed based on TIME phenotypes and gene clusters. Furthermore, we observed that the Gene G2 cluster was characterized by high TS, and Gene G1 was characterized by low TS, which correlated with poor and favorable prognosis of HCC, respectively. Correlation analysis showed that TS had a positive association with several clinicopathologic signatures **[**such as grade, stage, tumor (T), and node (N)] and known somatic gene mutations (such as *TP53* and *CTNNB1*). The prognostic value of the TS model was verified using external data sets.

**Conclusion:** We constructed a TS model based on differentially expressed genes and involving immune phenotypes and demonstrated that the TS model is an effective prognostic biomarker and predictor for HCC patients.

## Introduction

A tumor is a neoplasm caused by gene mutations and adaptation of resultant mutant cells to the microenvironment (1). The tumor immune microenvironment (TIME) is a complex and dynamic network system composed of immune cells, stromal cells, and immune matrix, and it is associated with tumorigenesis (2). Previous studies report that TIME plays an immune surveillance role by inhibiting tumor proliferation and preventing escape of tumor cells from immune system regulation (3), whereas some studies report that TIME could regulate the occurrence and development of tumors (4). More recently, studies have shifted to better understanding the association between TIME and tumorigenesis. Genomic analysis is a standard approach for studying the structure, function, evolution, and effects of genomes on organisms (5). Several methods have been established to act as a bridge between gene expression and immune cell components. Applying CIBERSORT, a computational method for predicting cell composition in tumor transcriptomes, may help map prognostic genes and leukocyte subsets within and across cancers, elucidate the effect of tumor heterogeneity on cancer prognosis, and identify diagnostic and therapeutic biomarker targets (6). xCell is also the usual method to calculate cell subsets of TIMEs from transcriptomes, which helps to understand the complex cellular heterogeneity in tumor tissues, improve existing treatments, identify predictive biomarkers, and develop new treatment strategies (7). Additionally, several studies have demonstrated that TIME regulates host and immune cell populations and, thus, can be used for tumor prognosis (8, 9). Notably, the immunosuppressive effect of TIME on tumors is regulated by immune cell components, such as T and B lymphocytes, macrophages, natural killer cells, and dendritic cells. However, changes to immune cell components, especially regulatory T cells and macrophages, promote tumor progression (10). These cell populations offer immunotherapeutic strategies and diagnostic and prognostic biomarkers for many solid tumor types, such as lung cancer, hepatocellular carcinoma (HCC), and breast and gastric cancers (11–14).

HCC is the leading cause of cancer-related morbidity and mortality worldwide, and most incidences are associated with cirrhosis related to chronic hepatitis virus infection (15). Currently, it is believed that immune escape contributes to the development of HCC caused by viral hepatitis infection—particularly hepatitis B virus (16). The liver is a key immune organ that plays a protective role by promoting immune tolerance. However, changes in immune tolerance signals or escape from immune surveillance in pathological conditions leads to HCC development (17). In addition, immunosuppressive cancer environments adversely affect innate and adaptive immunity function, resulting in HCC progression and metastasis (18). The TIME of the liver is a homeostatic system governed by effective regulatory mechanisms. However, ineffective TIME mechanisms, such as an imbalance involving immunosuppressive cell subsets, tumor signaling–mediated immune response enhancement, and antitumor immune fatigue, contribute to tumor progression (19). TIME-related immune cells, such as tumor-associated macrophages, tumor-associated neutrophils, tumor-infiltrating lymphocytes, regulatory T cells, CD8+ cytotoxic T lymphocytes, and natural killer cells, have been implicated in HCC pathogenesis. Moreover, TIME-based targets for HCC immunotherapy guide and improve the efficacy of various cancer therapies, particularly those that work by enhancing host antitumor immune responses (20). Immunotherapeutic approaches targeting immune checkpoints have been extensively studied to improve HCC immunotherapy effectiveness. Excessive immunomodulation, angiogenesis, inflammation, and communication between tumor cells and extracellular matrix can be targeted for HCC immunotherapy development (19). Previous studies report that TIME is important in the prediction of survival outcomes and in the evaluation of therapeutic efficacy (8, 9). However, immunomodulatory factors associated with HCC TIMEs have not been fully explored. Notably, the development of bioinformatics tools could facilitate efficient prediction of the composition of and change in TIMEs in tumors (21). Therefore, several studies have used bioinformatic tools to explore the clinical significance of TIME, the association of TIME and tumorigenesis, and the effect of immunotherapy on TIME (22, 23). However, the cellular and molecular features of TIME and their correlation with clinicopathological signatures in HCC have not been explored. The aim of this study, therefore, was to characterize TIME immune factors and explore their role in HCC.

In this study, gene expression data were retrieved from public databases and used to analyze 22 TIME immune cell components in 735 HCC patients. Furthermore, three immune phenotypes (TIME T1–3) were identified based on TIME to further evaluate associations among immune phenotypes, genomic characteristics (Gene G1–2), prognosis, and clinical features. We developed a TIME score (TS) model with good prognostic potential to be used as an immune biomarker for HCC (Supplementary Figure 1). Analysis of TIME landscape features may help in better understanding the role of immune factors in HCC TIME and provide new HCC immune biomarker and immunotherapy approaches.

## Methods

### Data Sources and Preparation

We searched public databases for gene expression data and clinical information regarding HCC patients. Six cohort data sets from The Cancer Genome Atlas (TCGA) and Gene Expression Omnibus (GEO) databases were downloaded. RNA-seq data of 424 HCC patients were downloaded from TCGA using the GDC API programmatic interface. Microarray data set GSE15654 containing data for 216 HCC patients, GSE76427 for 96 HCC patients, GSE14520 for 247 HCC patients and 241 normal controls, GSE36376 for 240 HCC patients and 193 normal controls, and GSE25097 for 269 HCC patients and 243 normal controls were downloaded from the GEO database. All samples from TCGA, GSE14520, GSE36376, and GSE25097 were randomly divided into training and validation sets. The RNA-seq read data from TCGA were preprocessed as follows: (1) HCC samples without clinical data and with overall survival (OS) <30 days were removed. (2) Normal tissue data were eliminated. (3) Genecode V22 annotation was used to transfer RNA-seq read data from fragments per kilobase million (FPKM) to transcripts per million (TPM). The distribution of TPM data was more similar to that of the microarray data than to the FPKM data. (4) Genes with a TPM expression value of 0 and that appeared in more than half of the samples were excluded. Microarray data from GEO were preprocessed as follows: (1) Normal tissue data were excluded, and thus, only primary tumor data were retained. (2) HCC samples without clinical data and OS <30 days were excluded. (3) The Bioconductor R package was used to map the chip probe to human gene SYMBOL.

### Calculation of Immune Cells in Time

The distribution of immune cells in TIME in HCC vs. normal control tissues was estimated using the CIBERSORT algorithm. Scores of each human immune cell in the three cohort data sets were calculated using the LM22 gene signature as a reference (the permutation parameter was 1,000) (6). The CIBERSORT algorithm is an anticonvolution support vector regression algorithm. This algorithm uses a set of minimum gene expression values (for 547 genes) to represent each cell type as a reference to infer the proportion of cell types in the data of a large number of tumor samples with mixed cell types. In addition, CIBERSORT can precisely and sensitively differentiate between 22 different human immune cells based on gene expression data. Some of these include T cells, macrophages, neutrophils, dendritic cells, B cells, and natural killer cells. Gene expression profiles were prepared using a standard annotation file, and the data were uploaded to the CIBERSORT website (http://cibersort.stanford.edu/), where the algorithm was executed using the LM22 gene signature and 1,000 permutations.

### Consensus Clustering of TIME-Infiltrating Cells

Unsupervised clustering of TCGA samples and tumor TIME-infiltrating cells was performed using the ConsensusClusterPlus algorithm based on the value obtained from TIME calculations. Euclidean distance calculation of similarity measures between clusters and K-means of unsupervised clustering were used to estimate the number of TIME clusters (24). The optimal number of clusters was determined by the cumulative distribution function (CDF) and the delta area and analyzed using the ConsensusClusterPlus R package with 1,000 repeats.

### Differential Gene Expression, Identification, and Clustering

Associations involving genes and TIME-infiltrating cells were explored by first dividing the genes into clusters based on the TIME-infiltrating cells. The DEseq2 tool was used to classify genes that were significantly differentially expressed and related to the TIME cluster in TCGA. Next, differentially expressed genes were selected by excluding genes with an expression value of 0 in >50% of samples. Furthermore, the non-negative matrix factorization (NMF) algorithm was used to perform unsupervised clustering (25). NMF is an effective method for identifying different molecular patterns and enabling class discovery, especially for biological information from cancer-related microarray data. In this study, we used the standard “Brunet” pattern for NMF analysis with 50 iterations (26). We set the number of clustering K-means from 2 to 10, determined the average contour width of the common member matrix through the NMF R package, and set the minimum member of each cluster to 10. The optimal clustering number was determined according to cophenetic, dispersion, and silhouette indicators.

### Construction of TIME Score Model

Before construction of the TIME score model, we identified common differentially expressed genes among the TIME clusters by dimensionality reduction. These genes were first subjected to univariate Cox analysis, after which a random forest algorithm was used to evaluate the importance of the genes using the R package (27). The random variable Mtry parameter was set for each partition, and the value with the lowest error rate was selected as the optimal Mtry value of the random Forest algorithm. Subsequently, we picked Ntree parameters according to the random Forest plot, and genes with cumulative importance >95% were chosen as candidates. Next, the K-means algorithm was used for cluster analysis through the ConsensusClusterPlus R package. Further, the Psych R package was applied to conduct principal component analysis (PCA). PCA uses dimensionality reduction technology to reduce multiple variables into a few principal components, which can reflect most attributes of the original variables (28). For each gene signature in the groups, 100 repeats were performed to obtain the optimal principal component numbers (PCs). The respective PC scores were calculated, and principal component 1 (PC1) scores of each cluster were selected as the signature score. Subsequently, Cox multivariate regression analysis was used to construct a prognosis risk model for each group. A TIME score = ∑PC1^*^β formula was used to define the TIME score model, in which β is the multivariate regression coefficient of each group, and PC1 is the score of each group.

### Statistical Analysis

A forest plot was created using the Forest plot R package, based on univariate Cox regression analysis results of each data set. A univariate Cox proportional hazard risk regression model was used to calculate univariate risk ratio. The statistical significance of normally and non-normally distributed data was calculated using Student's *t*-test, and two independent variables were analyzed using Wilcoxon's sign rank test. Non-parametric testing of three or more sets of data was performed using Kruskal–Wallis tests. The least absolute shrinkage and selection (LASSO) and random-forest analyses were used to select suitable immune cell fractions. These immune cell risk scores were used to construct diagnostic models based on the coefficients of each selected marker through a logistic regression algorithm. HCC patients were assigned to high- and low-risk groups using the median value or were adjusted by Z-scores such that >0 and <0 were defined as high- and low-risk groups, respectively. The Kaplan–Meier (KM) method was used to plot survival curves for estimating survival rates of patients, and statistical differences among means were compared using the log-rank test. Immune and stromal scores of each sample were calculated using the ESTIMATE tool employing the R package. Receiver operating characteristic (ROC) curves, which were generated with Package pROC, were used to determine the sensitivity and specificity of the KM analysis. A diagram showing the association between TIME scores and gene biology was developed using the Corrplot R package. NetworkD3 R packages were used to construct an alluvial diagram of TIME clusters with different gene clusters and survival outcomes. ComplexHeatmap R packages were used to depict the mutational landscape of genes. HCC patients were classified into high- and-low risk groups based on median TIME scores for survival analysis. The limma R package was used to analyze differential expression of TIME cluster genes, and functional enrichment was performed using the cluster profile R package. All statistical analyses in this study were conducted using either the R package or SPSS software, and *P* < 0.05 was considered statistically significant.

## Results

### Identification of TIME-Infiltrating Cells and Classification of TIME Phenotypes

**Figure 1:**
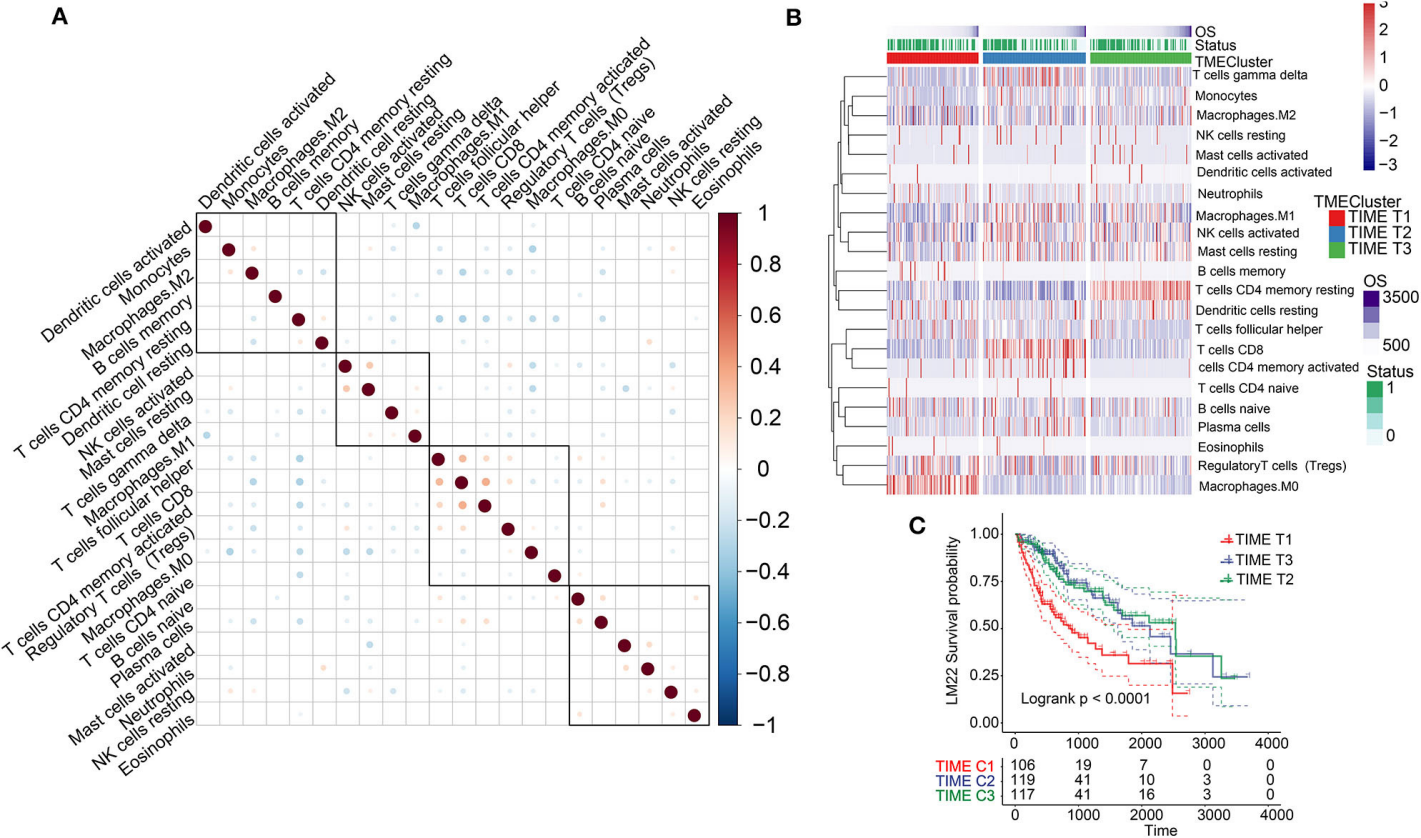
TIME-infiltrating cells and classification of TIME. **(A)** Correlations among 22 types of immune cells in TIME. Brown and blue nodes represent positive and negative correlations, respectively. The larger the node, the stronger the correlation. **(B)** Heat map illustrating results of unsupervised clustering based on TIME phenotypes. **(C)** Kaplan–Meier analysis for three TIME phenotypes.

Analysis of the TIME-infiltrating cell component by the CIBERSORT algorithm revealed 22 immune cell classifications. These included B cells, T cells, natural killer (NK) cells, macrophages, and dendritic cells (DCs). Correlation analysis further grouped the 22 categories into four groups (Figure 1A). These four groups were positively correlated, implying communication among the 22 immune cell types. Furthermore, we carried out univariate Cox analysis to test the prognostic factor of the 22 immune cell types. Forest plots showed that follicular helper T cells (*P* = 0.038) and M0 macrophages (*P* = 0.008) were unfavorable prognostic markers [hazard ratio (HR) >1], whereas CD8+ T cells (*P* = 0.021) and resting CD4+ memory T cells (*P* = 0.031) were favorable prognostic markers (HR <1) (Supplementary Figure 2A). We performed unsupervised clustering of 735 tumors from three HCC cohorts with TIME-matched cell expression profiles (Supplementary Figure 3A). The clustering results revealed three phenotypes (TIME T1–3) of TIME-infiltrating cells based on optimal K = 3 and verification of CDF and delta area (Figure 1B, Supplementary Figures 3B,C). Additionally, we observed that TIME T1 was characterized by high levels of regulatory T cells (Tregs) and M0 macrophages. TIME T2 was primarily associated with CD8+ T cells and activated CD4+ memory T cells, and TIME T3 was characterized by resting CD4+ memory T cells, resting DCs, and activated NK cells. To verify the value of infiltrating immune cells as biomarkers for HCC, we compared the composition of infiltrating immune cells between HCC and normal tissue in 4 data sets (TCGA, GSE14520, GSE36376, GSE25097) to understand their distribution and roles as potential HCC biomarkers. We identified significant differences in the composition of immune-infiltrating cells between HCC and normal tissue across the four data sets. Notably, Treg and M0 macrophage numbers were significantly higher in HCC tissue compared with normal tissue, and CD8+ T and resting CD4+ memory T cell levels were significantly lower in HCC tissue (Supplementary Figure 4A). The distribution of infiltrating immune cells in HCC tissue across the clinical features showed that key immune cells, including M0 macrophages, resting CD4+ memory T cells, M1 macrophages, activated NK cells, and CD8+ T cells constituted the majority of such cells (Supplementary Figure 4B). In addition, we analyzed associations involving key immune cells and clinical features (tumor-node- metastasis (TNM), stage, and grade). Apart from no statistical significance in some analyses, M0 macrophage and Treg scores were higher in advanced pathological stages (Supplementary Figures 5A,B). In contrast, resting CD4+ memory T cells and CD8+ T cell scores decreased in advanced pathological stages (Supplementary Figures 5C,D). These results reveal the components of immune infiltrating cells in HCC and indicate that Tregs, M0 macrophages, CD8+ T cells, resting CD4+ memory T cells, and activated CD4+ memory T cells are key biomarkers in HCC.

KM survival analysis based on the three phenotypes identified revealed that TIME T1 was associated with poor prognosis, whereas TIME T2 and TIME T3 exhibited favorable HCC prognosis (*P* < 0.0001) (Figure 1C). The distribution of TIME-infiltrating cells among the three phenotypes was analyzed using the Kruskal–Wallis test (Supplementary Figure 2B). TIME T1 was characterized by high levels of Tregs and M0 macrophages, and the levels of M1 and M2 macrophages in TIME T1 were lower compared with the levels in TIME T2 and T3 because M1 and M2 are regarded as classically and alternatively activated macrophages, respectively. In different immune microenvironments, three types of macrophages can be activated and transformed into subsets with different molecular and functional characteristics. In addition, TIME T2 exhibited higher levels of CD8+ T cells and activated CD4+ memory T cells, and TIME T3 was characterized by high numbers of resting CD4+ memory T cells, resting DCs, and NK cell activation.

However, it is not clear whether one or several specific immune cells could be used as HCC biomarkers. Therefore, we conducted random forest (Supplementary Figure 4C) and LASSO (Supplementary Figure 4D) analysis of the 4 data sets (TCGA, GSE14520, GSE36376, GSE25097). The two analysis methods revealed 8 possible HCC markers (Tregs, M0 macrophages, CD8+ T cells, resting CD4+ memory T cells, activated CD4+ memory T, activated NK cells, activated mast cells, and T cell follicular helpers). Furthermore, a diagnostic model based on the risk score involving these immune cells was constructed using a logistic regression method. The results show that the risk scores for HCC patients are significantly higher than those for normal controls among the four data sets (Supplementary Figure 4E). ROC analysis verified the high accuracy of the diagnostic model based on such immune cell risk scores to distinguish HCC patients from normal controls (Supplementary Figures 4F,G). In summary, our results illustrate that TIME-infiltrating cells and phenotypes with different patterns of immune cellular components could be used as potential HCC prognostic biomarkers.

### Identification of Gene Clusters and Analysis of Biological Function

**Figure 2:**
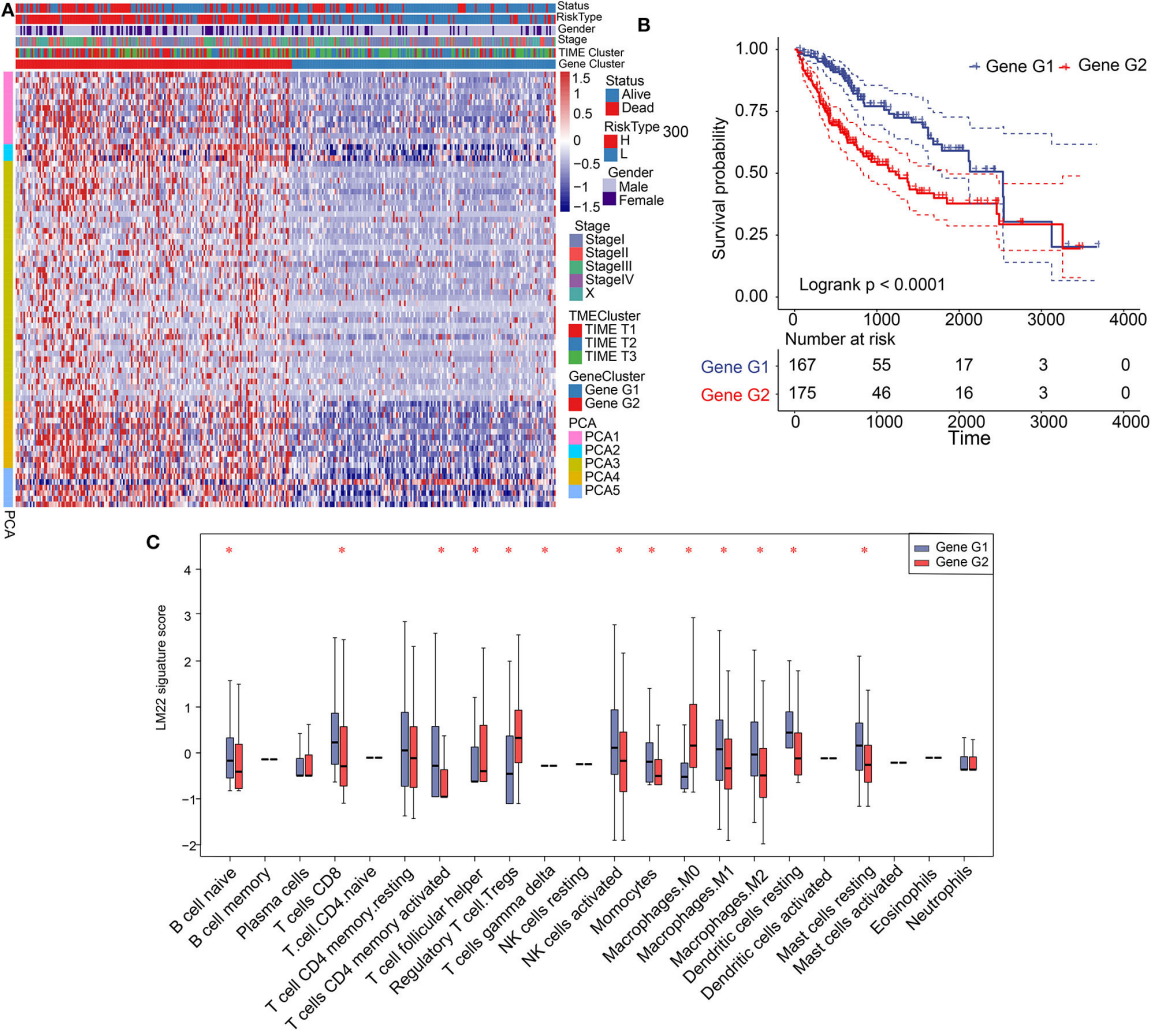
Construction of gene clusters and association between TIME signature and TIME gene patterns. **(A)** Heat map showing results of unsupervised clustering based on gene cluster classification. **(B)** Kaplan–Meier analysis of 2 gene clusters. **(C)** Distribution and expression of 22 types of immune cells in the 2 gene clusters. *P < 0.05.

Significant differences in patient prognosis involving TIME T1 and TIME T2/T3 were observed. Therefore, we analyzed differentially expressed genes (DEGs) between TIME T1 and TIME T2 and TIME T1 and TIME T3. In total, we identified 432 DEGs between TIME T1 and TIME T2 and TIME T1 and TIME T3 (Supplementary Figure 6A). After being screened by the NMF algorithm (Supplementary Figure 3D), the 432 DEGs were reduced to 365 and classified into two clusters (Gene G1–2) based on the optimal K = 2 (Supplementary Figure 6B). Unsupervised clustering analysis of the 365 DEGs grouped HCC patients into two classes (Figure 2A). We observed that most Gene G1 members were associated with TIME T2/T3 and were characterized by low risk, and most Gene G2 members were associated with TIME T1 and were characterized by high risk. KM analysis showed that Gene G1 and Gene G2 were associated with good and poor prognoses, respectively (*P* < 0.0001) (Figure 2B). We used the alluvial diagram to illustrate relationships involving the three phenotypes (TIME T1–3) and the two clusters (Gene G1–2) as well as their living status (Supplementary Figure 6C). Notably, the distribution of TIME-infiltrating cells among the two gene clusters (Figure 2C) was consistent with the three phenotypes (Supplementary Figure 2B). These findings indicate that Gene G2 is characterized by high levels of Tregs and M0 macrophages, and Gene G1 is characterized by CD8+ T cells and activated CD4+ memory T cells, resting CD4+ memory T cells, resting DCs, and NK cells activation. In summary, classification of patients based on genomic clusters is consistent with TIME phenotype groups.

Gene G1 and Gene G2 represented significant differences in the distribution of TIME-infiltrating cells and prognosis; therefore, we further investigated differences in cellular biological functions involving these genes. We conducted Kyoto Encyclopedia of Genes and Genomes (KEGG) analysis using biological pathways. We determined that Gene G1 is associated with immune processes, such as T cell receptor signaling pathways, Th1 and Th2 cell differentiation, immune system function, and complement activation. In contrast, most members of Gene G2 are involved in tumorigenesis processes, including the P53 signaling pathway, PI3K-Akt signaling, hepatocellular carcinoma, and apoptosis (Supplementary Figures 7A,B). Therefore, we constructed a network of genes and pathways that revealed a regulatory relationship between immune-related pathways in Gene G1 and tumorigenesis-related pathways in Gene G2, and these pathways interacted through overlapping genes (Supplementary Figures 7C,D). The results reveal that Gene G1 and Gene G2 are associated with immune and tumorigenesis functions, respectively. Therefore, these findings may explain the favorable prognosis of Gene G1 and the poor prognosis of Gene G2 cases.

### Establishment of TIME Score Model and Analysis of Clinical Signature Associations

**Figure 3:**
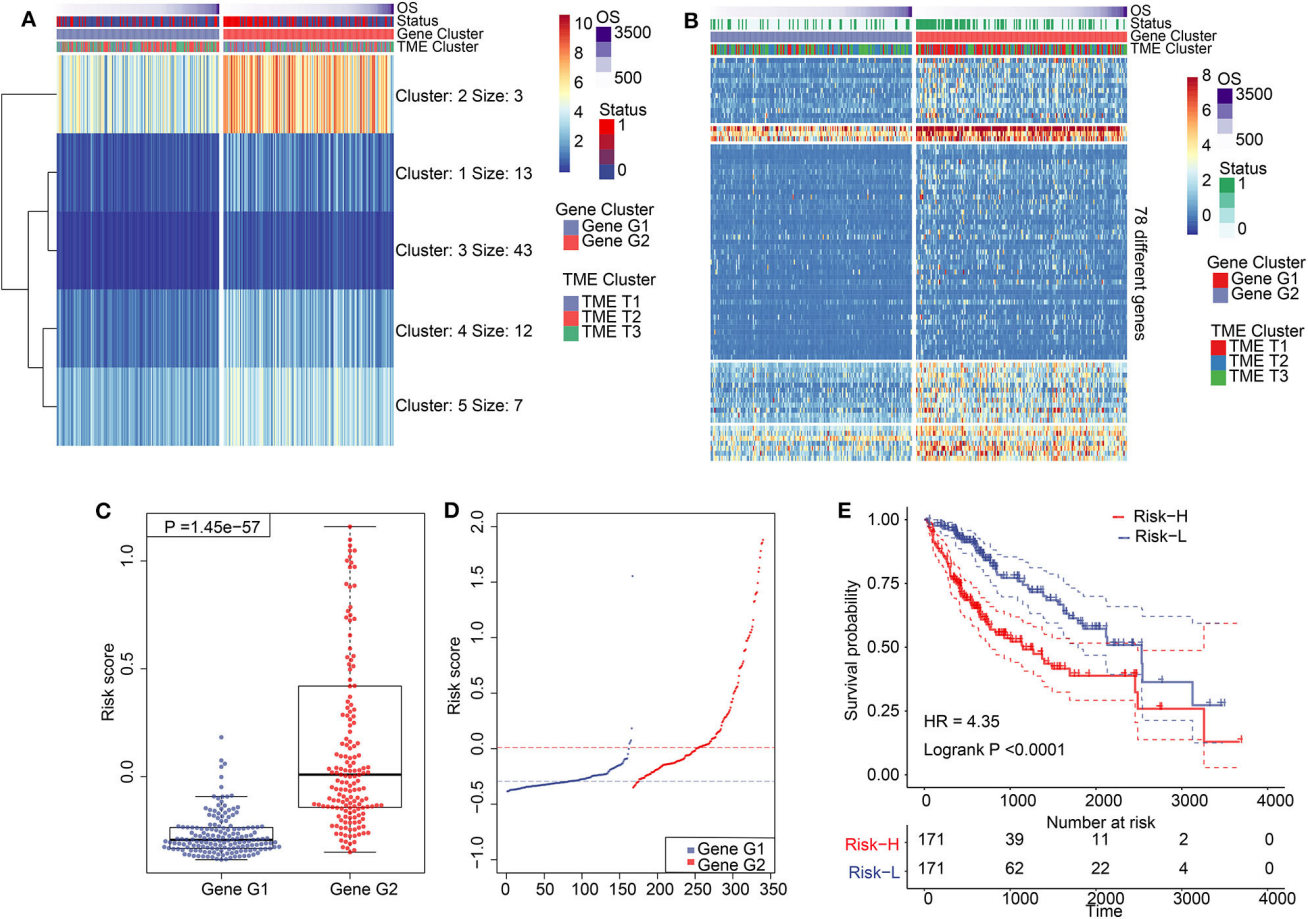
Construction of TIME score model and its characteristics. **(A)** K-means clustering results of 78 differentially expressed genes. **(B)** A clustering heat map of 78 differentially expressed genes. **(C)** Comparation of TS between Gene G1 and Gene G2. **(D)** Distribution of TS between Gene G1 and Gene G2. **(E)** Kaplan–Meier analysis for high TS and low TS.

**Figure 4:**
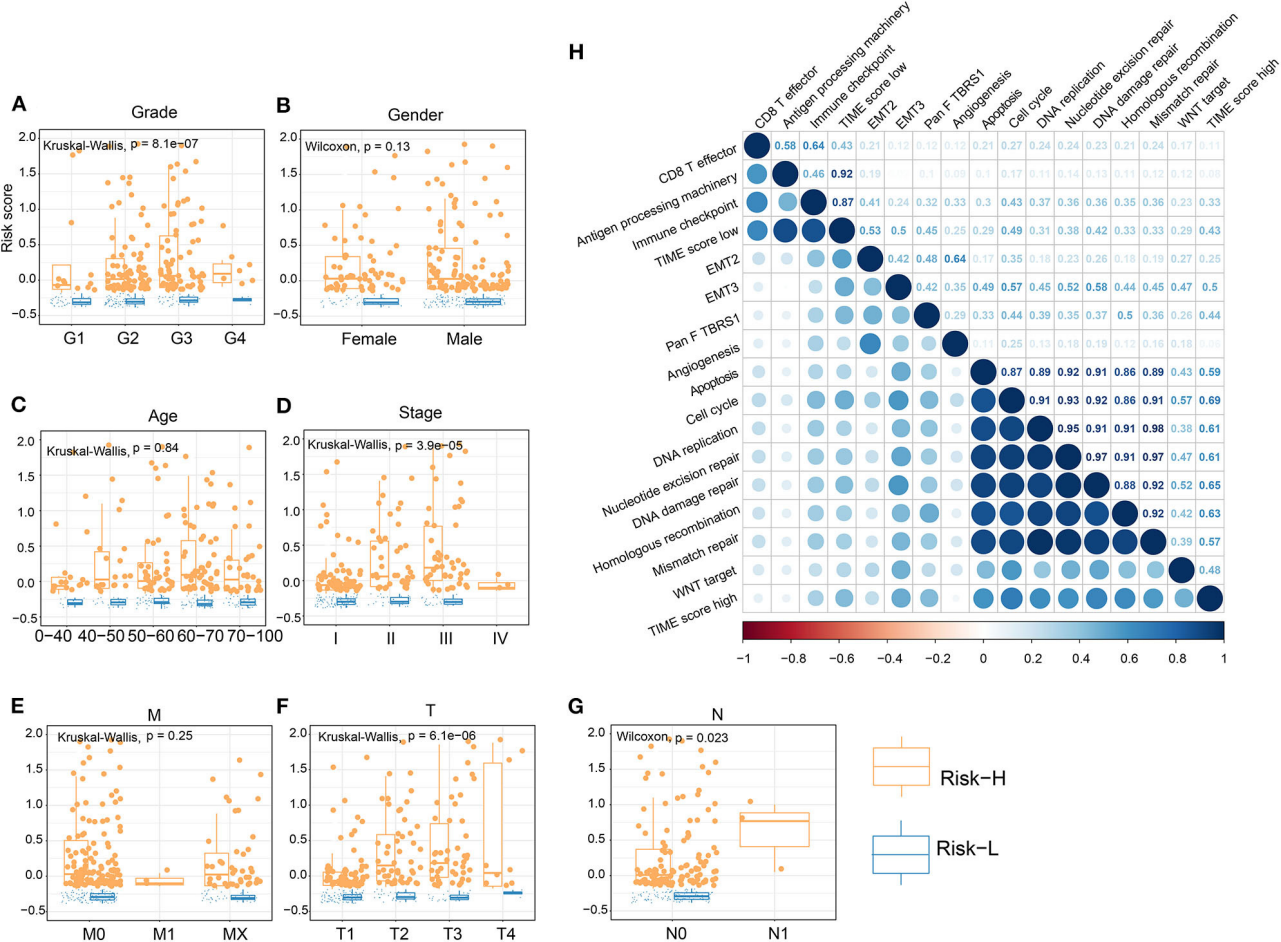
Association of TS with clinical characteristics and biological signatures. **(A–G)** Association of TS with clinical characteristics, including grade, gender, age, stage, M, T, and N. **(H)** Association involving TS and known biological signatures. (J) Forest plot showing results of multivariate Cox analysis for TS and clinical characteristics.

We performed dimension reduction to reduce redundant genes. A total of 117 DEGs were identified after univariate Cox analysis. Analysis of 117 DEGs using the random forest algorithm (Supplementary Figure 3E) identified 78 DEGs. Analysis of the biological functions of the 78 DEGs by Gene Ontology (GO) indicated that these genes are involved in cell differentiation, cell–cell junction, inflammatory responses, and antibiotic responses (Supplementary Figure 8A). KEGG pathway analysis of the 78 genes enriched in the immune system indicated HCC, Th1, and Th2 cell differentiation; immune responses; innate complement; and Toll-like receptor signaling pathways (Supplementary Figure 8B). These results show that the 78 DEGs are implicated in tumorigenesis and immune responses. Based on clustering analysis, the 78 DEGs were classified into five groups, which we assigned the signatures 1–5 (S1–5). There were 13, 3, 43, 12, and 7 DEGs in the S1, S2, S3, S4, and S5 groups, respectively (Figure 3A). Among these, S2 was a high-expression group, S1 and S3 were low-expression groups, and S4 and S5 were intermediate expression level groups. A heat map of the 78 DEGs is presented in Figure 3B, which is consistent with the clustering plot. Furthermore, we carried out PCA analysis to construct a TIME score model according to the PC1 scores of each group. In addition, we constructed a prognostic score model, which we termed the TS score model. On comparing Gene G1 and Gene G2, we found that the TS score of Gene G2 was significantly higher than that of Gene G1 (Figures 3C,D). In addition, we performed ESTIMATE algorithm processing to compare stromal and immune scores across TIME1–3 and observed significant increases in stromal and immune scores in TIME1. Although no statistical significance was observed, the ESTIMATE score for TIME1 was higher than that for TIME1 and TIME2 (Supplementary Figure 9). HCC patients were assigned to a high TS or low TS score using a median value (−0.185). High and low TS scores were associated with poor and good prognosis, respectively (*P* < 0.0001) (Figure 3E). These results were consistent with the KM analysis of gene clusters (Figure 2B), in which Gene G2 indicated poor prognosis compared with Gene G1. We further analyzed the association between TS scores and clinical signatures, and the results showed that the grade, tumor (T), node (N), and stage classifications exhibited significantly different TS scores (*P* < 0.05) (Figures 4A,D,F,G). However, we did not observe any clinical significance between metastasis (M), gender, and age (*P* > 0.05) (Figures 4B,C,E). To study the role of immune factors involving TS scores, we investigated potential associations between TS scores and previously studied immune genes (14). In this analysis, immune-activated genes (*TBX21, CXCL9, GZMA, GZMB, PRF1, IFNG, TBX2, TNF*, and *CD8A*), immune checkpoint genes (*PDCD1, CTLA4, LAG3, PDCD1LG2, CD274*, and *HAVCR2*), and transforming growth factor/epithelial-mesenchymal transition genes (TGF/EMT) (*VIM, ACTA2, COL4A1, TGFBR2, ZEB1, CLDN3, SMAD9*, and *TWIST1*) were used. The results reveal differences in gene-expression patterns between different gene clusters, TS scores, and TIME phenotypes (Supplementary Figure 10A). However, we found that TS scores were closely associated with immune genes. Furthermore, we explored the correlation between known signatures [EMT, immune checkpoints, tumorigenesis, biological processes (cell cycle, angiogenesis, mismatch repair)] and TS scores to describe the function of our TS score model. We observed that high TS scores were associated with tumorigenic features, such as apoptosis, cell cycle, DNA replication, mismatch repair, and WNT targeting. On the other hand, low TS scores were associated with factors implicated in immune activation, including CD8+ T effector, antigen-processing machinery, and immune checkpoint steps (Figure 4H). Furthermore, when the TS model was tested as a variable signature by Cox regression, the forest plot showed that the TS model was an independent HR prognostic factor, with a more substantial HR value than other clinical signatures (Supplementary Figure 10B). All of these results demonstrate that the TS model is a robust feature and can, therefore, be used to predict patient HCC prognosis. Furthermore, these findings reveal that the TS model is associated with several clinical signatures.

### Comparison Between TS Model and Known Signatures

**Figure 5:**
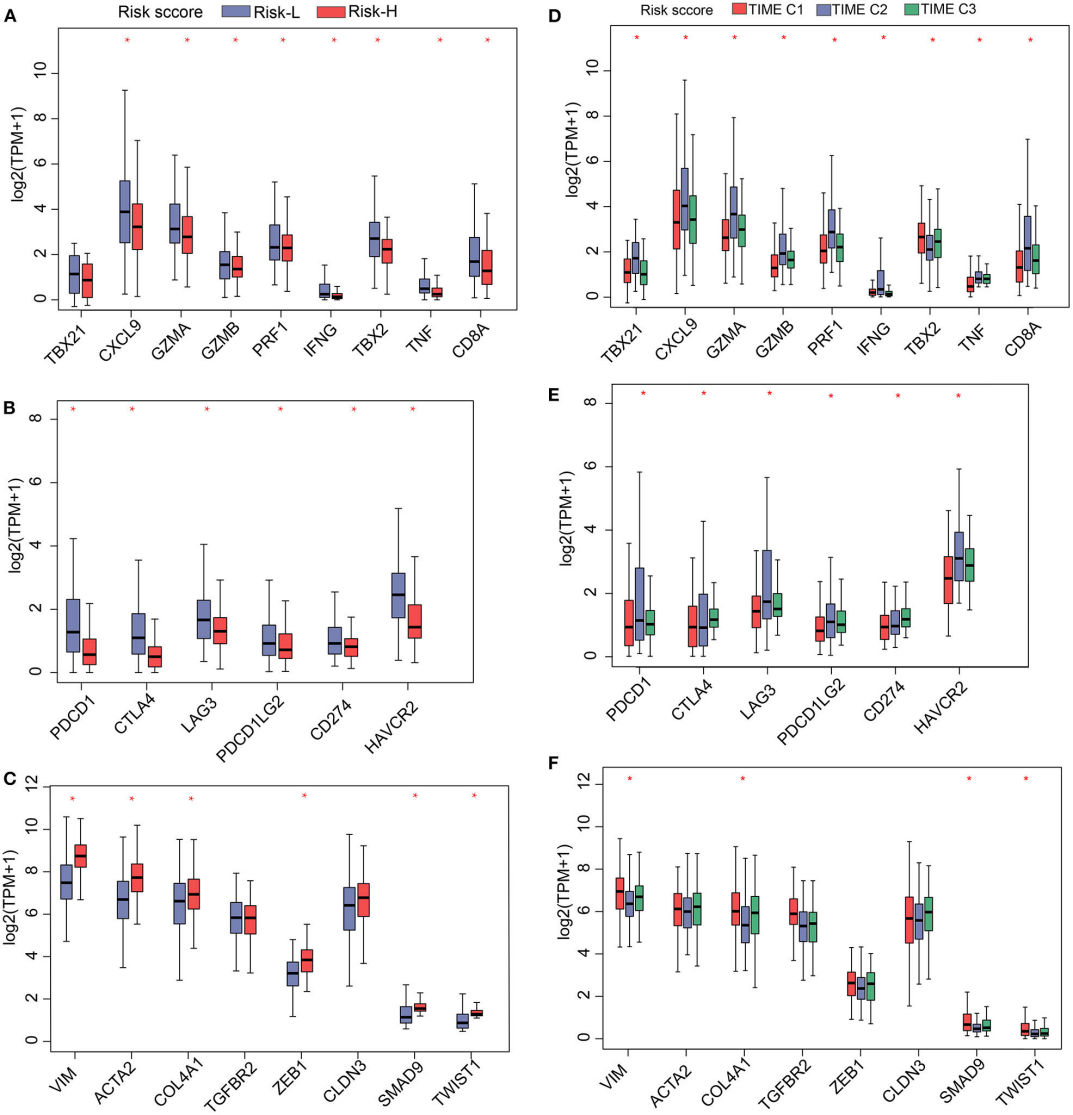
Gene expression profiles of **(A)** immune activation, **(B)** immune checkpoint proteins, and **(C)** TGF/EMT in high and low TS. Gene-expression profiles of **(D)** immune activation, **(E)** immune checkpoint proteins, and **(F)** TGF/EMT in 3 TIME phenotypes. *P < 0.05.

Having shown that the TS model is a useful prognostic biomarker, we sought to understand associations involving the identified TS model and known HCC signatures. Therefore, we analyzed the expression of immune-activated genes, immune checkpoint genes, and TGF/EMT genes in the high and low TS score categories. The results indicate that low TS scores are associated with elevated expression of immune-activated and immune checkpoint genes (Figures 5A,B), whereas high TS scores are characterized by high expression of TGF/EMT genes (Figure 5C). Additionally, we evaluated the expression of immune-activated genes, immune checkpoint genes, and TGF/EMT genes in the TS scores of TIME T1–3 and observed that the expression of immune-activated and immune checkpoint genes in TIME T2 and TIME T3 was elevated compared with that in TIME T1 (Figures 5D,E). In contrast, the expression of TGF/EMT genes in TIME T1 was higher than in TIME T2/T3 (Figure 5F). These findings suggest that low TS scores related to TIME T2/T3 are associated with immune-activated and immune checkpoint genes, which trigger immune functions to suppress tumor development. Therefore, low TS scores may represent a favorable HCC prognostic marker. However, high TS scores related to TIME T1 were associated with TGF/EMT genes, which are linked to tumorigenesis, which results in unfavorable HCC prognosis. Furthermore, we explored the distribution of known somatic mutations involving gene expression and analyzed relationships between TS scores and these genes. Using the Fisher's exact test (*P* < 0.05), we compared known somatic gene alterations exhibiting significant differences in mutation frequency between high and low TS score groups. A total of 49 variants were found to be associated with TS scores (Supplementary Figure 11). *TP53*, an anticancer gene (29), for instance, was mainly distributed in high TS scores. However, mutated *TP53* lost intrinsic cancer inhibitory function and exhibited poor patient prognosis. The *CTNNB1* gene causes cancer, and mutated *CTNNB1* was distributed in both high and low TS scores. A previous study reports that *TP53* mutation is implicated in tumor development, and *TP53* can be targeted with HCC checkpoint inhibitors for immunotherapy development (30). Other genes, such as *RB1, TLL1*, and *PIK3CA*, are implicated as important factors in genetic alterations in HCC (31, 32). *RB1* is one of the most significantly mutated genes in HCC and is related to proteogenomic phenotype classification and involved in distinct features in metabolic reprogramming, microenvironment dysregulation, and cell proliferation (33). Genome-wide association studies have found that *TLL1* variants are associated with HCC after hepatitis C virus infection eradication (34). A previous study reports that blood-derived circulating tumor DNA markers, such as *PIK3CA* with frequent alteration, may be key biomarkers in diagnosis of advanced HCC and for HCC molecular diagnosis (35). This study presents a new perspective for exploring the immune mechanisms involved in immunotherapy of tumors.

### Validation of the TS Model

**Figure 6:**
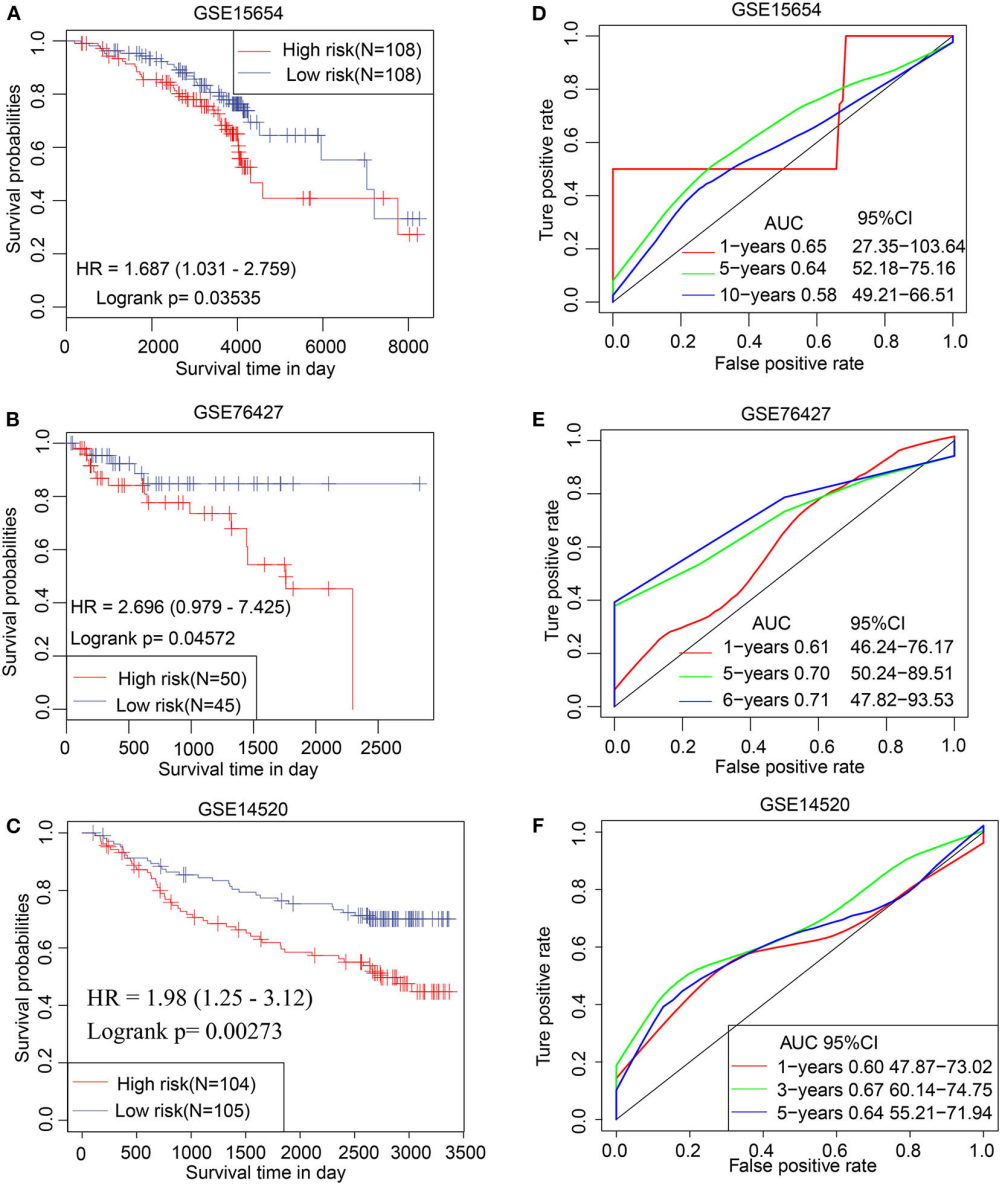
Validation of TS model involving 3 independent data sets. Kaplan–Meier analysis for high and low TS in **(A)** GSE15654, **(B)** GSE76427, and **(C)** GSE14520 data sets. ROC curves of **(D)** GSE15654, **(E)** GSE76427, and **(F)** GSE14520 illustrating the predictive value of TS.

The prognostic efficacy of the TS model was validated using the GSE15654, GSE76427, and GSE14520 data sets by KM analysis. The results indicate that a high TS score is significantly associated with poor prognosis, whereas a low TS score is significantly associated with favorable prognosis in the GSE15654 (*P* = 0.03535), GSE76427 (*P* = 0.04572), and GSE14520 (*P* = 0.00273) data sets (Figures 6A–C). The sensitivity of KM analysis was verified by ROC analysis. The results of ROC analysis show that the TS model is a predictive biomarker for HCC patients (GSE15654: AUC of 1 year = 0.65, 5 years = 0.64, 10 years = 0.58; GSE76427: AUC of 1 year = 0.61, 5 years = 0.70, 6 years = 0.71; GSE14520: AUC of 1 year = 0.60, 3 years = 0.67, 5 years = 0.64) (Figures 6C–F). These results further suggest that the TS model is an effective HCC predictor of prognostic signature and has defined replicability for different data sets

## Discussion

In this study, data obtained by comprehensive analysis of TIME-infiltrating cells and relevant genes were used to construct a TS model. This model accurately predicted the prognosis of HCC patients. Systematic analysis revealed that high TS scores were associated with poor prognosis, immune suppression, and tumorigenesis, whereas low TS scores were correlated with favorable prognosis, immune activation, and immune checkpoint progression. Liver cells are highly immune-tolerant. This is because immune cells in the liver form an immune-tolerance state that protects against autoimmune-induced damage. Carcinogenic factors, such as persistent viral infection, compromise immune tolerance or balance rendering immune cells unable to clear carcinogenic factors (17, 36). In the early stages of tumor growth, immune suppression decreases immune surveillance (37). Thus, the primary target of tumor immunotherapies, such as PD-1/PD-L1, is to activate and restore immune function for optimal ablation of tumor cells (38). In low-TS groups, our results show that immune activation correlates with better prognosis, suggesting that immune activation inhibits HCC tumorigenesis. This is consistent with a previous study in which key genes and tumor-associated leukocytes were identified to predict the prognosis of cancer patients and their responses to targeted therapy (39). However, the significance of our study involves not only the analyzed composition of infiltrating immune cells in HCC and classified HCC patients based on molecular phenotypes, but also systematically associated TIME phenotypes and gene clusters with genomic characteristics and clinical and pathologic features. In so doing, we identified biomarkers with potential clinical application. These biomarkers were used to construct a TS model that could predict the prognosis of HCC patients.

Analysis of TIME-infiltrating cells and phenotypes reveale that M0 macrophages were unfavorable factors assigned to TIME T1, whereas CD8+ T cells and CD4+ T cells were favorable factors assigned to TIME T2/T3. These results are consistent with those from previous research in which T cells and macrophages are reported to inhibit and promote HCC, respectively (40). M0 macrophages are undifferentiated macrophages with the potential to transform into specific subtypes of macrophages (41). Different subtypes of liver macrophages exhibit diverse ontogeny, differentiation, and function, especially Kupffer cells and tumor-associated macrophages (TAMs) (42). TAMs play an important role in the occurrence, development, invasion, metastasis, immune evasion, and angiogenesis in HCC (43). Kupffer cells enhance virus-mediated inflammation, causing liver cirrhosis and HCC (44). Liver macrophages exhibit highly variable phenotypes that are modulated by signals derived from the liver microenvironment (42). We hypothesized that M0 macrophages may stimulate the production of TAM and Kupffer cells in the presence of carcinogenic factors and, thus, promote inflammation and suppress immunity leading to HCC development. Compared to normal tissues, M0, M1, and M2 macrophage levels were generally higher in HCC cells. Macrophages are classically polarized into activated macrophages (M1) and alternatively activated macrophages (M2) under the stimulation of different immune microenvironments (45). The induction of M1 from M0 macrophages and the mutual transformation of M1 and M2 macrophages modulates tumorigenesis (46). Our research reveals that enrichment of macrophages in HCC predicts poor prognosis. T cells (CD8+ T cells and CD4+ T cells) are the key immune cells that kill tumor cells by activating the immune system (47). For this reason, novel immunotherapies, such as PD-1 and PD-L1, have been designed to modulate the activity of T cells. The role of PD-1/PD-L1 is to block the binding of tumor cells and T cells, allowing guardian T cells to identify and eliminate tumor cells (48). Activation of T cells in TIME inhibits tumor cells, and this may explain why CD8+ T cells and CD4+ T cells in TIME T2/T3 were associated with good prognosis.

Integrated analysis identified our TS model to be a prognostic biomarker associated with previously studied immune genes. In line with prior studies, upregulation of genes associated with immune activation and immune checkpoint proteins correlates with better prognosis, whereas upregulation of genes associated with TGF/EMT correlates with poorer prognosis (14, 49). In this study, we find that low TS reflects good prognosis, and high TS indicates poor prognosis, suggesting that the TS model is a robust prognostic biomarker. Further analysis of TS scores revealed that elevated TS was accompanied with tumorigenesis signatures, such as cell cycle, DNA replication, mismatch repair, and WNT targeting, whereas low TS was characterized by activation of CD8+ T cell effector and antigen-processing machinery. These results are in agreement with the prevailing knowledge that pathological division of cells is the basis of tumorigenesis (50) and that CD8+ T cells can kill tumor cells by facilitating antigen processing (51). In addition, we observed that our TS model was associated with several known somatic mutations, involving *TP53* and *CTNNB1*. Alterations of these somatic genes may inactivate tumor suppressor genes and cause mutations in proto-oncogenes, resulting in tumorigenesis (52). Therefore, our study contributes to the identification of immunotherapeutic targets aimed at inhibiting pathways involved in tumorigenesis.

Compared with previous studies regarding TIME and HCC (53), this investigation was performed using a large number of HCC samples. Moreover, unlike previous studies (54), which focused only on the function of immune cells in TIME, we comprehensively mapped the landscape of interactions involving TIME-infiltrating cells, genes, and clinicopathological features. Using bioinformatics algorithms, we constructed a TS model and assessed the association between the TS model and clinicopathological features. We find that the TS model is significantly associated with grade, T, N, and, stage. Moreover, we find that the prognostic value of the TS model is superior to that of other clinical signatures. Previous studies find a correlation between clinicopathological classification and immune response, and this implies that an immune response–related signature can be used for clinicopathological classification (55). Yutaka et al. analyze the immune microenvironment of HCC tissues and intratumor heterogeneity. They observe that several immune subtypes are associated with poor differentiation of HCC (55). In a study by Sia et al., HCC is subcategorized into 2 subclasses based on immune-specific characteristics; adaptive and exhausted immune responses. Notably, the exhausted immune subclass exhibited immunosuppression due to overexpression of TGF-1-regulated genes, which led to poor prognosis (40). Our study provides a better understanding of the TIME, upon which general histological/molecular classification of HCC based on TIME, can be achieved.

## Conclusions

In conclusion, this study reveals that immune characteristics of TIME modulate the pathogenesis of HCC. A TS model was constructed based on TIME phenotypes and gene clusters, which exhibited robust prognostic predictive value for HCC patients. We also reveal promising candidate immune-based biomarkers for diagnosis, prognosis, and immunotherapy in HCC.

## Data Availability Statement

The datasets presented in this study can be found in online repositories. The names of the repository/repositories and accession number(s) can be found in the article/Supplementary Material.

## Ethics Statement

The study was approved by the Clinical Research Ethics Committee of College of Medicine, Zhejiang University. Written informed consent for participation was not required for this study in accordance with the national legislation and the institutional requirements.

## Author Contributions

HD: research design and funding acquisition. WC, XZ, KB, YD, HZ, and JX: acquisition, interpretation, and analyses of data. WC, XZ, KB, and HZ: manufacture of figures. WC: writing of manuscript and article language modification. All authors contributed to the article and approved the submitted version.

## Conflict of Interest

The authors declare that the research was conducted in the absence of any commercial or financial relationships that could be construed as a potential conflict of interest. The handling editor declared a shared affiliation, though no other collaboration, with the authors.

## Supplementary Material

Supplementary Figure 1 A flow chart of the study.

Supplementary Figure 2 **(A)** Forest plot illustrating results of univariate Cox analysis for the 22 types of immune cells investigated. **(B)** Heat map showing results of unsupervised clustering based on TIME phenotypes.

Supplementary Figure 3 Algorithms used to classify TIME phenotypes. **(A)** Consensus matrix of TIME-infiltrating cells for each K (2–5) with the corresponding heat maps. **(B)** CDF analysis for consensus cluster analysis. **(C)** Delta area curves for consensus cluster analysis. **(D)** Algorithms used to classify non-negative matrix factorization of gene clusters. **(E)** Distribution of random forest error rates across tree parameters. **(F)** Multidimensional scaling plot for Gene G1 and Gene G2 data. **(G)** Random Forest plot for significant DEGs with mean decrease in gini index (blue) and mean decreased accuracy (red).

Supplementary Figure 4 Comparison of immune cells in HCC and normal control samples. **(A)** Comparison of immune cell fraction differences in HCC and normal tissues. **(B)** Distribution of immune cell fraction across the clinical characteristics of HCC tissues. **(C)** A random forest plot showing the multidimensional scale plot of adjacent matrix. **(D)** LASSO regression model illustrating misclassification errors across different quantitative variables. **(E)** Comparison of immune scores between HCC and normal tissue control samples. ROC analysis of the diagnostic model in **(F)** training and **(G)** validation set.

Supplementary Figure 5 Association involving key immune cells and clinical features. Comparison of immune scores of **(A)** M0 macrophages, **(B)** Tregs, **(C)** resting CD4+ memory T cells, and **(D)** CD8+ T cells across TNM, stage, and grade.

Supplementary Figure 6 **(A)** Venn diagram illustrating intersection of differentially expressed genes across TIME T1 and TIME T2 and TIME T1 and TIME T3. **(B)** Consistency matrix heat map of NMF algorithm. **(C)** An alluvial diagram showing the association between 3 TIME phenotypes and 2 gene clusters.

Supplementary Figure 7 Biological function of genes in 2 gene clusters. The main KEGG pathways of **(A)** Gene G1 and **(B)** Gene G2. Relationship network of genes and pathways in **(C)** Gene G1 and **(D)** Gene G2.

Supplementary Figure 8 **(A)** GO and **(B)** KEGG analyses of the 78 identified differentially expressed genes.

Supplementary Figure 9 Comparison of **(A)** stromal, **(B)** immune, and **(C)** ESTIMATE scores across TIME1–3 groups.

Supplementary Figure 10 **(A)** Heat map showing genes associated with immune activation, immune checkpoint proteins, and TGF/EMT. **(B)** A Forest plot showing results of multivariate Cox analysis for TS and clinical characteristics.

Supplementary Figure 11 Association between TS and somatic mutations.
