# Supplementary figures and images for: Comprehensive Study of Tumor Immune Microenvironment and Relevant Genes in Hepatocellular Carcinoma Identifies Potential Prognostic Significance
Source: Front Oncol. 2020 Sep 24;10:554165. doi: 10.3389/fonc.2020.554165 (PMC7541903; doi:10.3389/fonc.2020.554165)

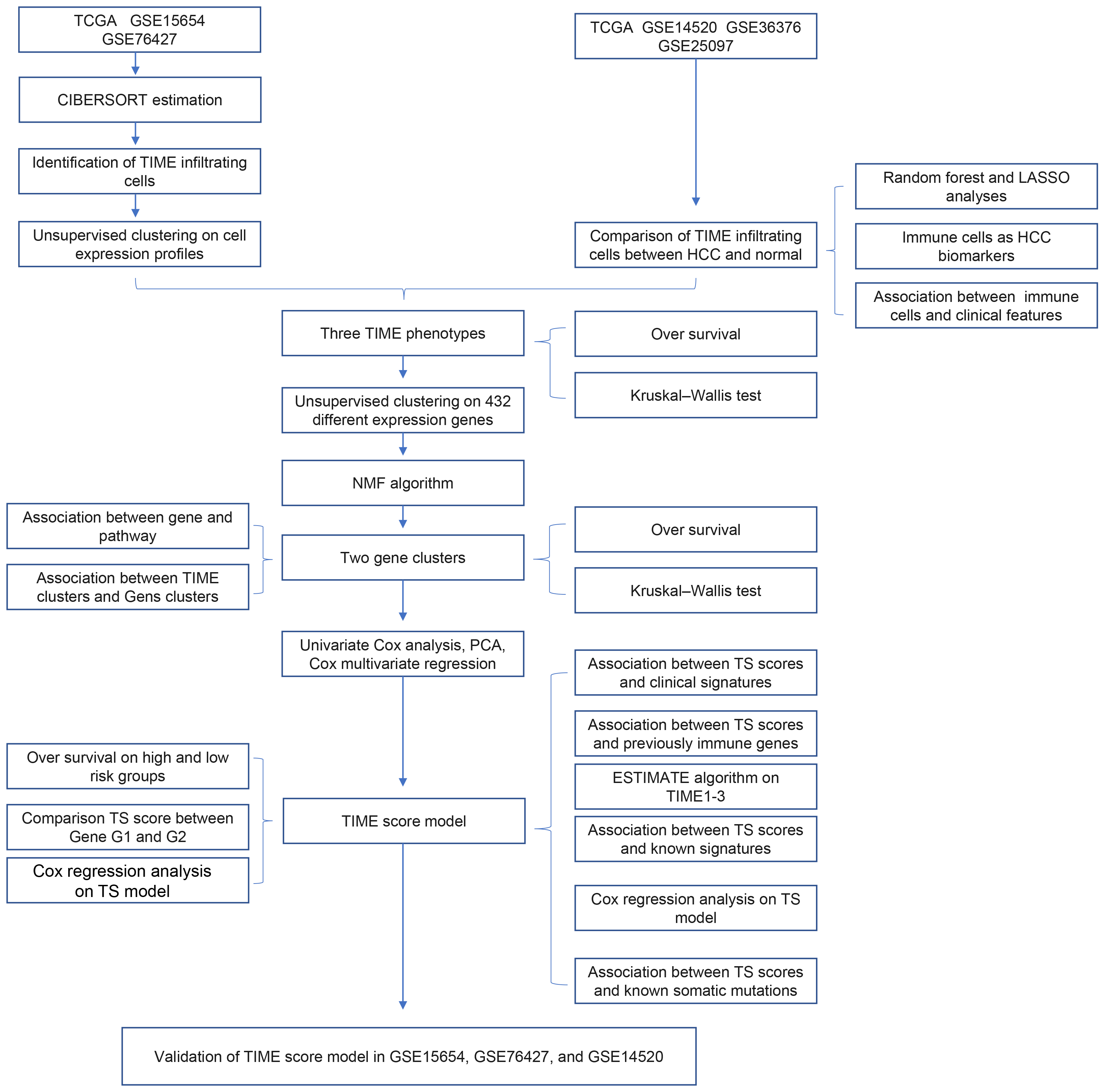

Supplement: Supplementary Figure 1 — A flow chart of the study. [file Image_1.tif]

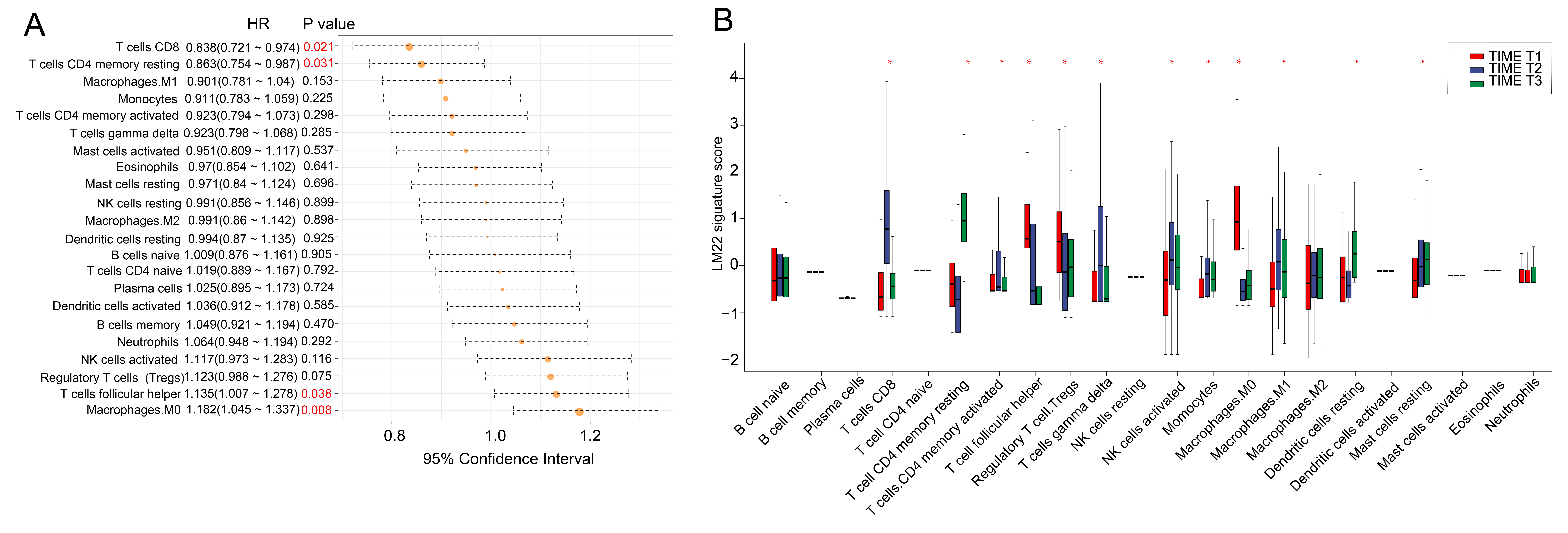

Supplement: Supplementary Figure 2 — (A) Forest plot illustrating results of univariate Cox analysis for the 22 types of immune cells investigated. (B) Heat map showing results of unsupervised clustering based on TIME phenotypes. [file Image_2.tif]

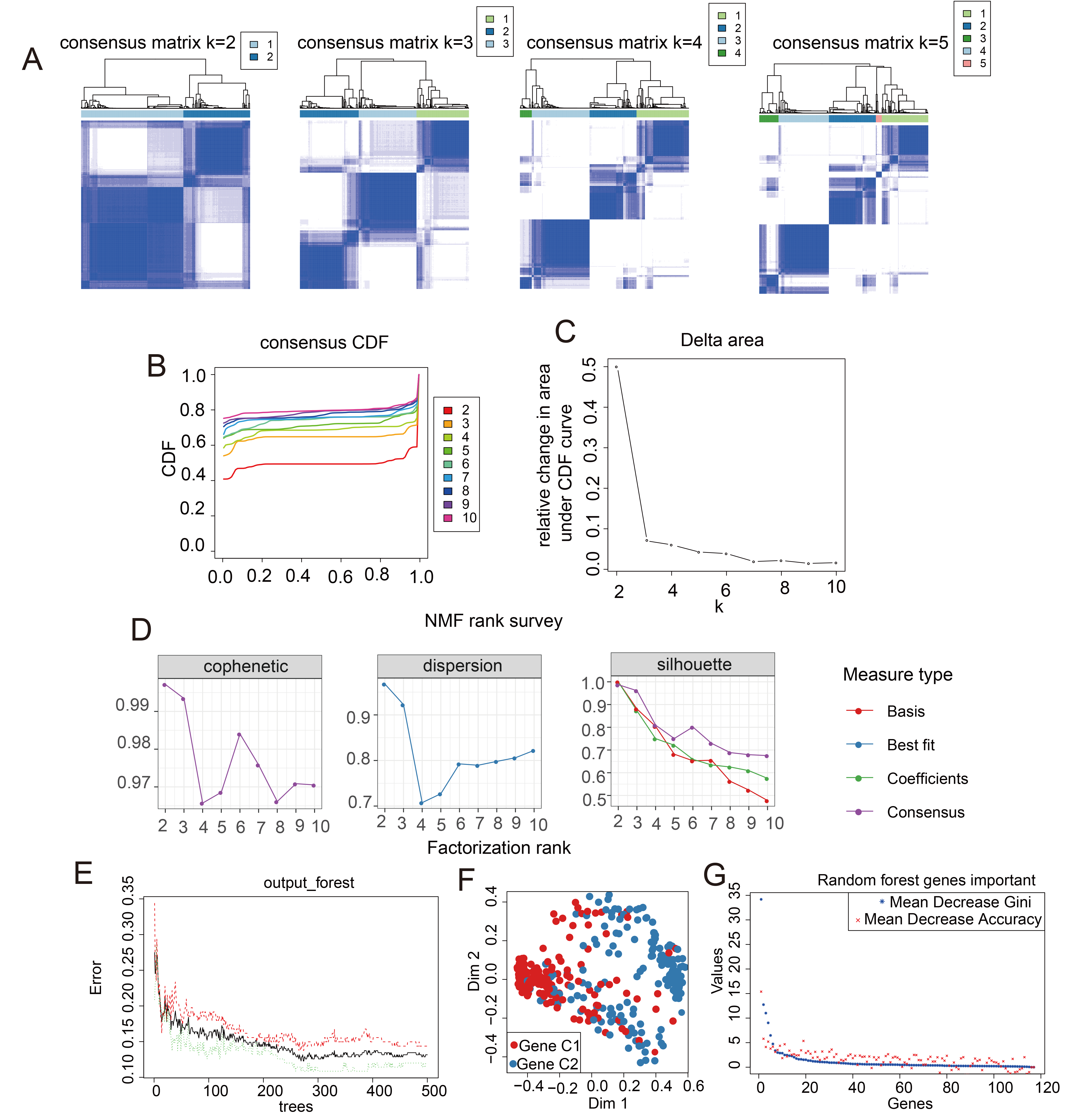

Supplement: Supplementary Figure 3 — Algorithms used to classify TIME phenotypes. (A) Consensus matrix of TIME-infiltrating cells for each K (2–5) with the corresponding heat maps. (B) CDF analysis for consensus cluster analysis. (C) Delta area curves for consensus cluster analysis. (D) Algorithms used to classify non-negative matrix factorization of gene clusters. (E) Distribution of random forest error rates across tree parameters. (F) Multidimensional scaling plot for Gene G1 and Gene G2 data. (G) Random Forest plot for significant DEGs with mean decrease in gini index (blue) and mean decreased accuracy (red). [file Image_3.tif]

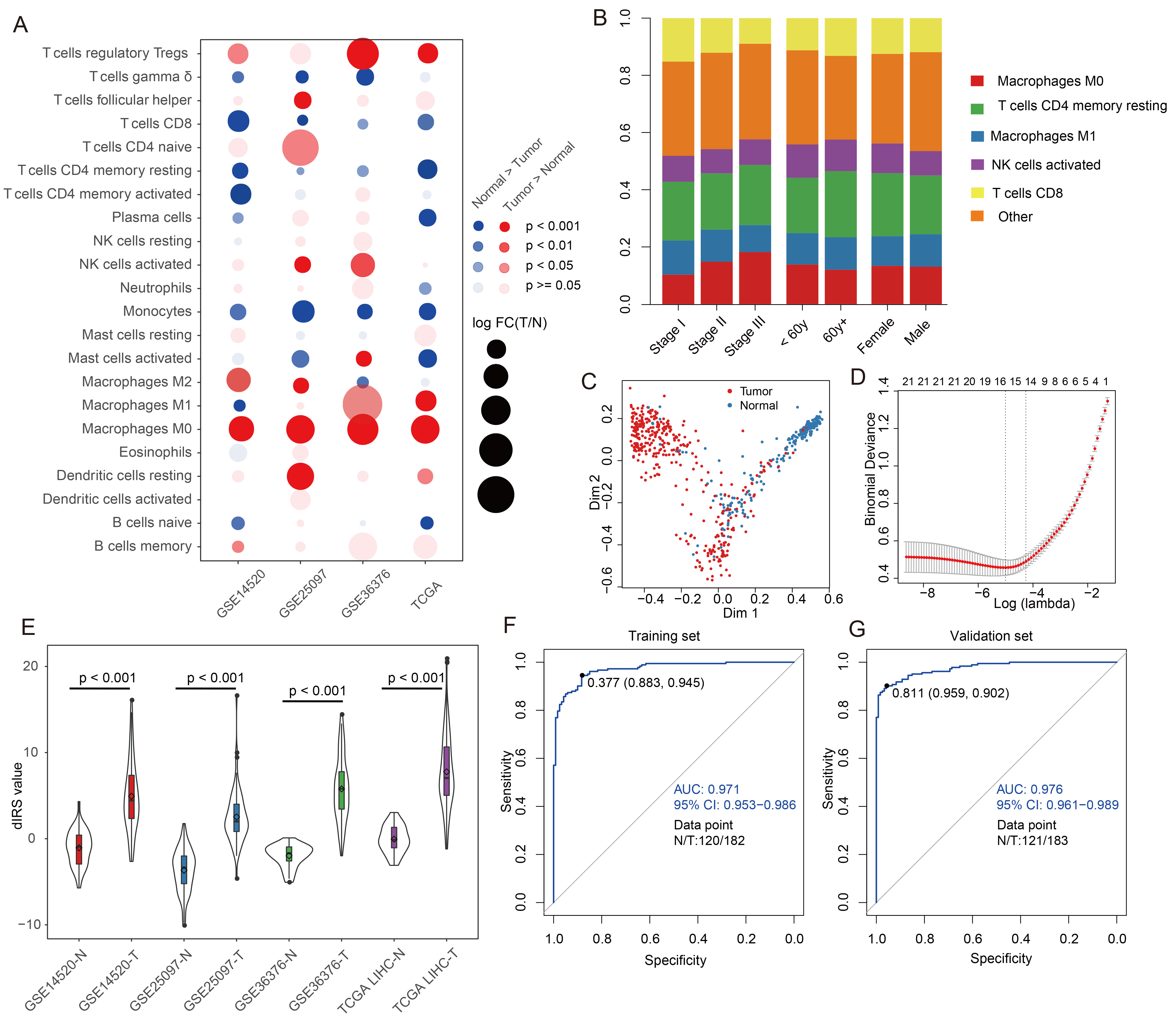

Supplement: Supplementary Figure 4 — Comparison of immune cells in HCC and normal control samples. (A) Comparison of immune cell fraction differences in HCC and normal tissues. (B) Distribution of immune cell fraction across the clinical characteristics of HCC tissues. (C) A random forest plot showing the multidimensional scale plot of adjacent matrix. (D) LASSO regression model illustrating misclassification errors across different quantitative variables. (E) Comparison of immune scores between HCC and normal tissue control samples. ROC analysis of the diagnostic model in (F) training and (G) validation set. [file Image_4.tif]

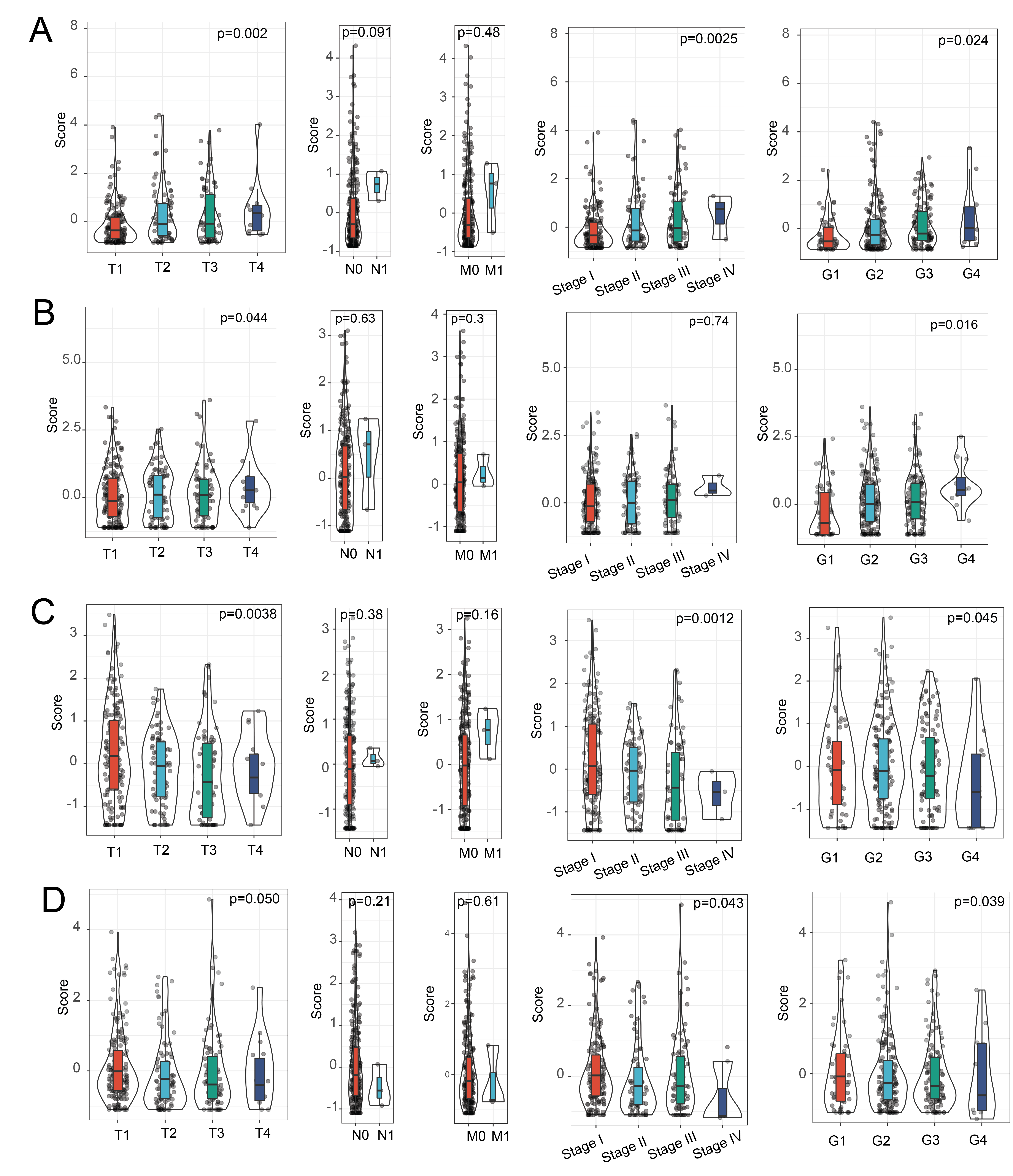

Supplement: Supplementary Figure 5 — Association involving key immune cells and clinical features. Comparison of immune scores of (A) M0 macrophages, (B) Tregs, (C) resting CD4+ memory T cells, and (D) CD8+ T cells across TNM, stage, and grade. [file Image_5.tif]

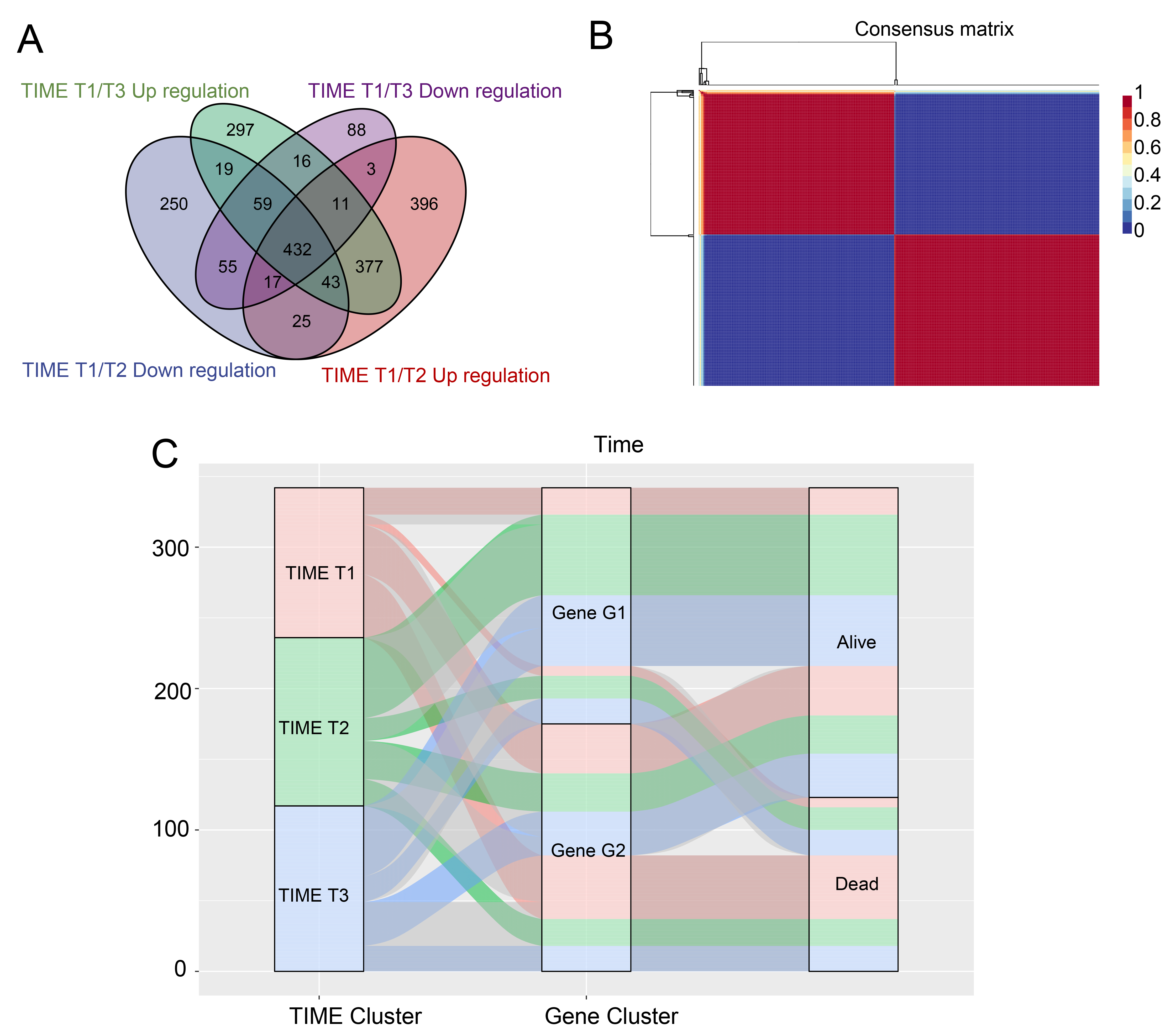

Supplement: Supplementary Figure 6 — (A) Venn diagram illustrating intersection of differentially expressed genes across TIME T1 and TIME T2 and TIME T1 and TIME T3. (B) Consistency matrix heat map of NMF algorithm. (C) An alluvial diagram showing the association between 3 TIME phenotypes and 2 gene clusters. [file Image_6.tif]

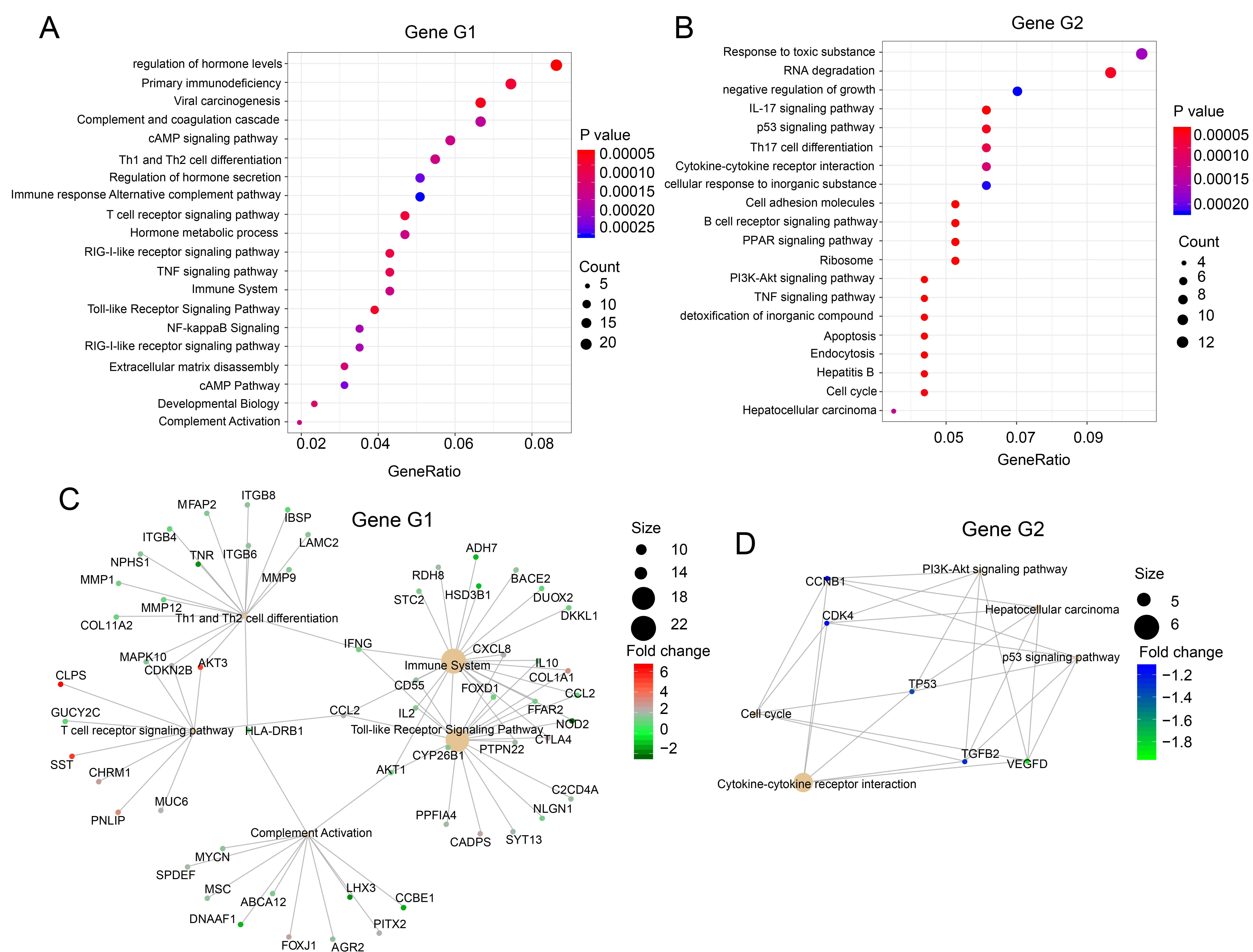

Supplement: Supplementary Figure 7 — Biological function of genes in 2 gene clusters. The main KEGG pathways of (A) Gene G1 and (B) Gene G2. Relationship network of genes and pathways in (C) Gene G1 and (D) Gene G2. [file Image_7.tif]

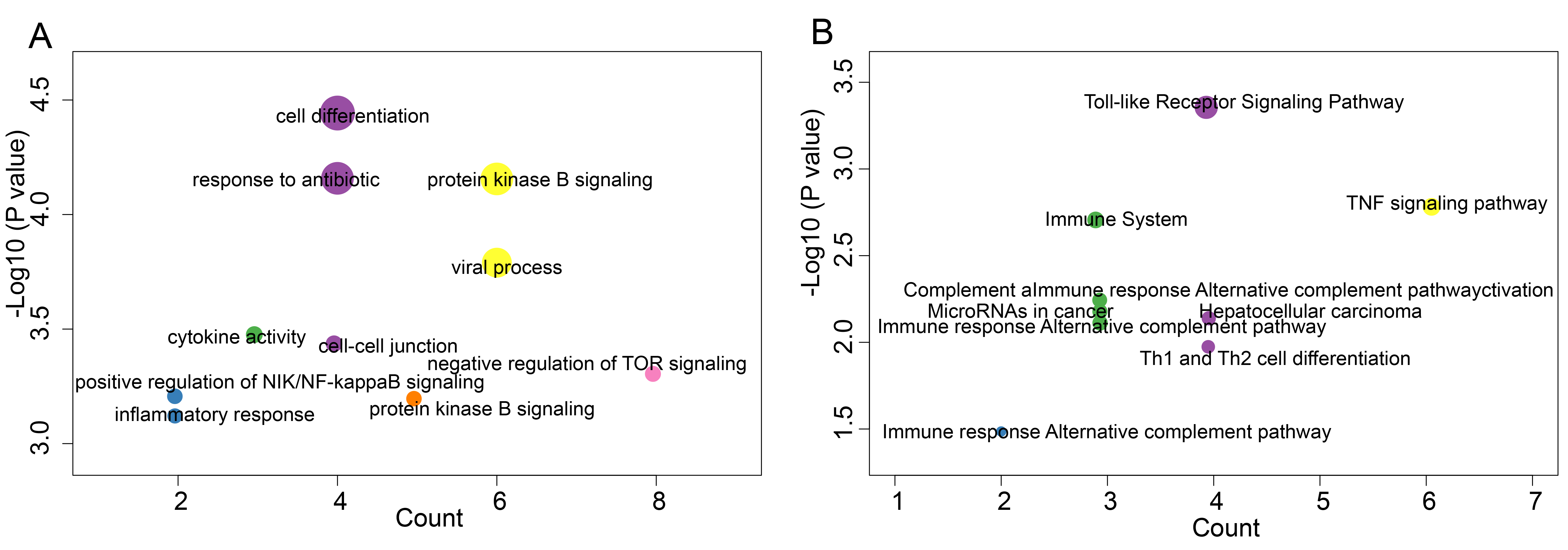

Supplement: Supplementary Figure 8 — (A) GO and (B) KEGG analyses of the 78 identified differentially expressed genes. [file Image_8.tif]

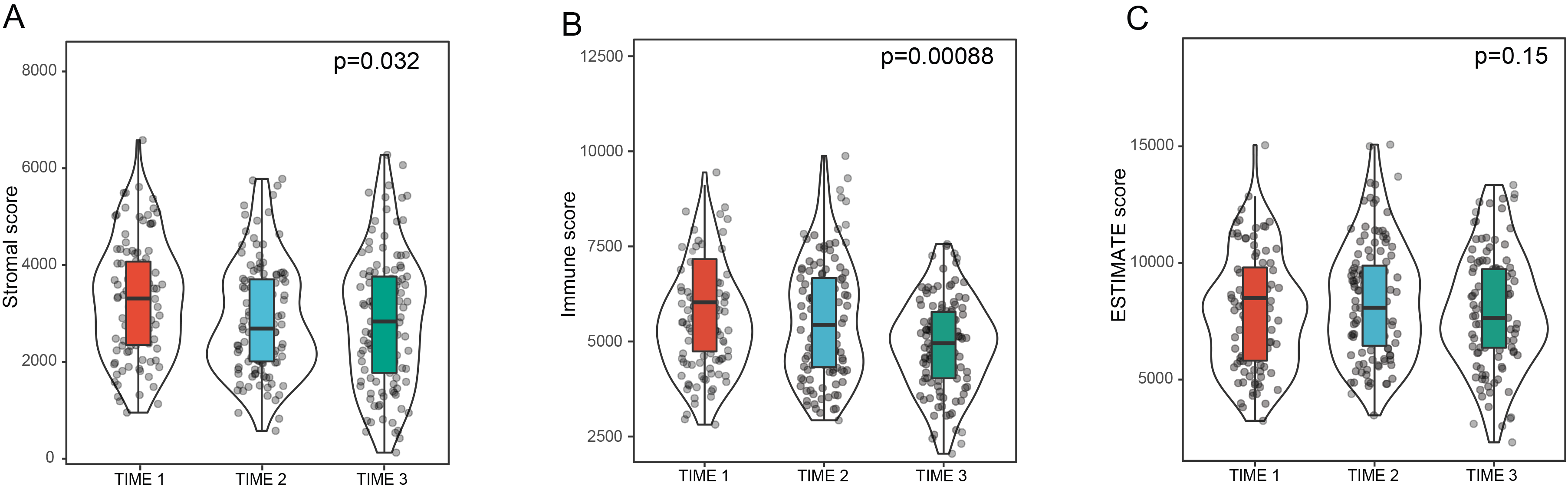

Supplement: Supplementary Figure 9 — Comparison of (A) stromal, (B) immune, and (C) ESTIMATE scores across TIME1–3 groups. [file Image_9.tif]

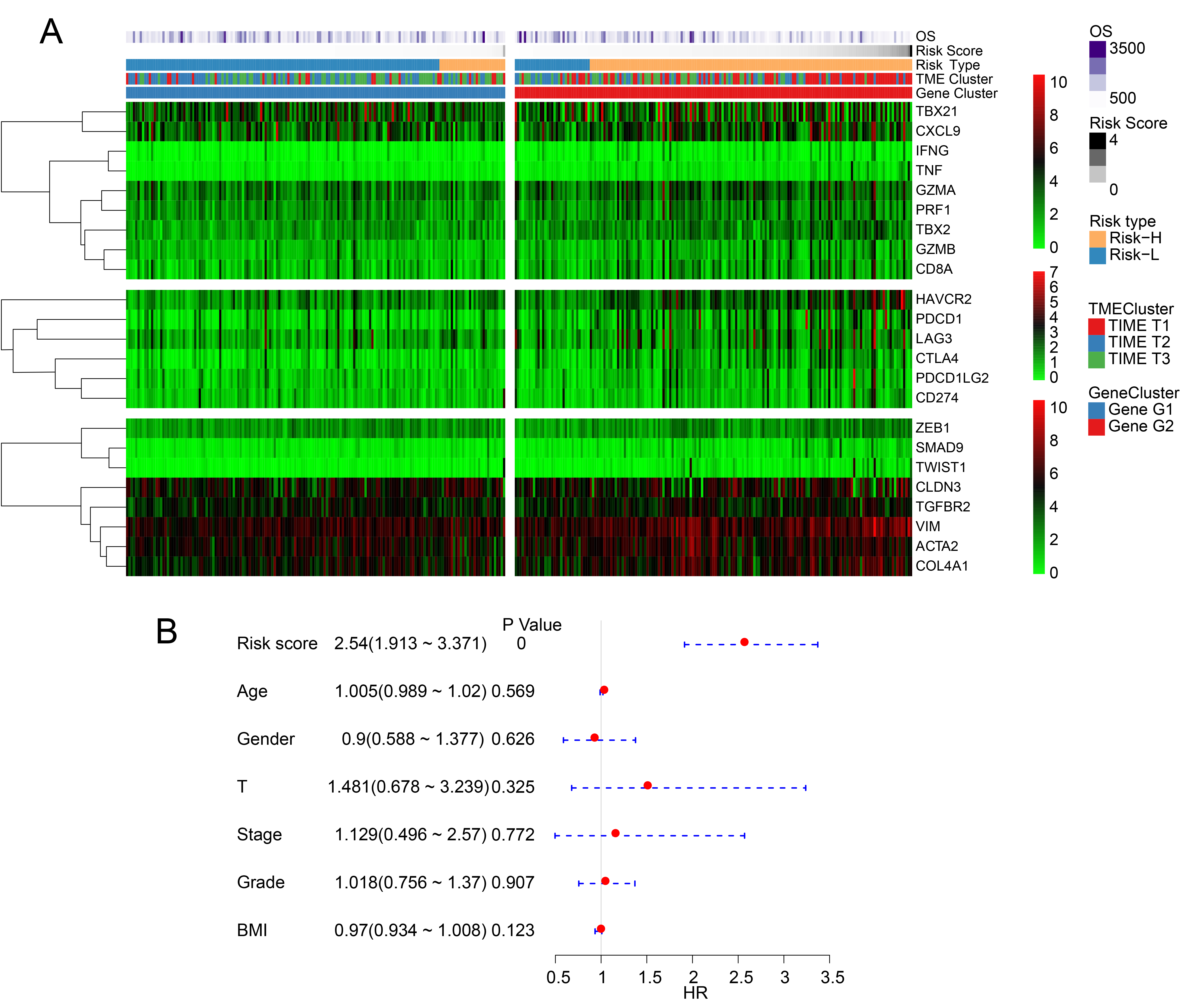

Supplement: Supplementary Figure 10 — (A) Heat map showing genes associated with immune activation, immune checkpoint proteins, and TGF/EMT. (B) A Forest plot showing results of multivariate Cox analysis for TS and clinical characteristics. [file Image_10.tif]

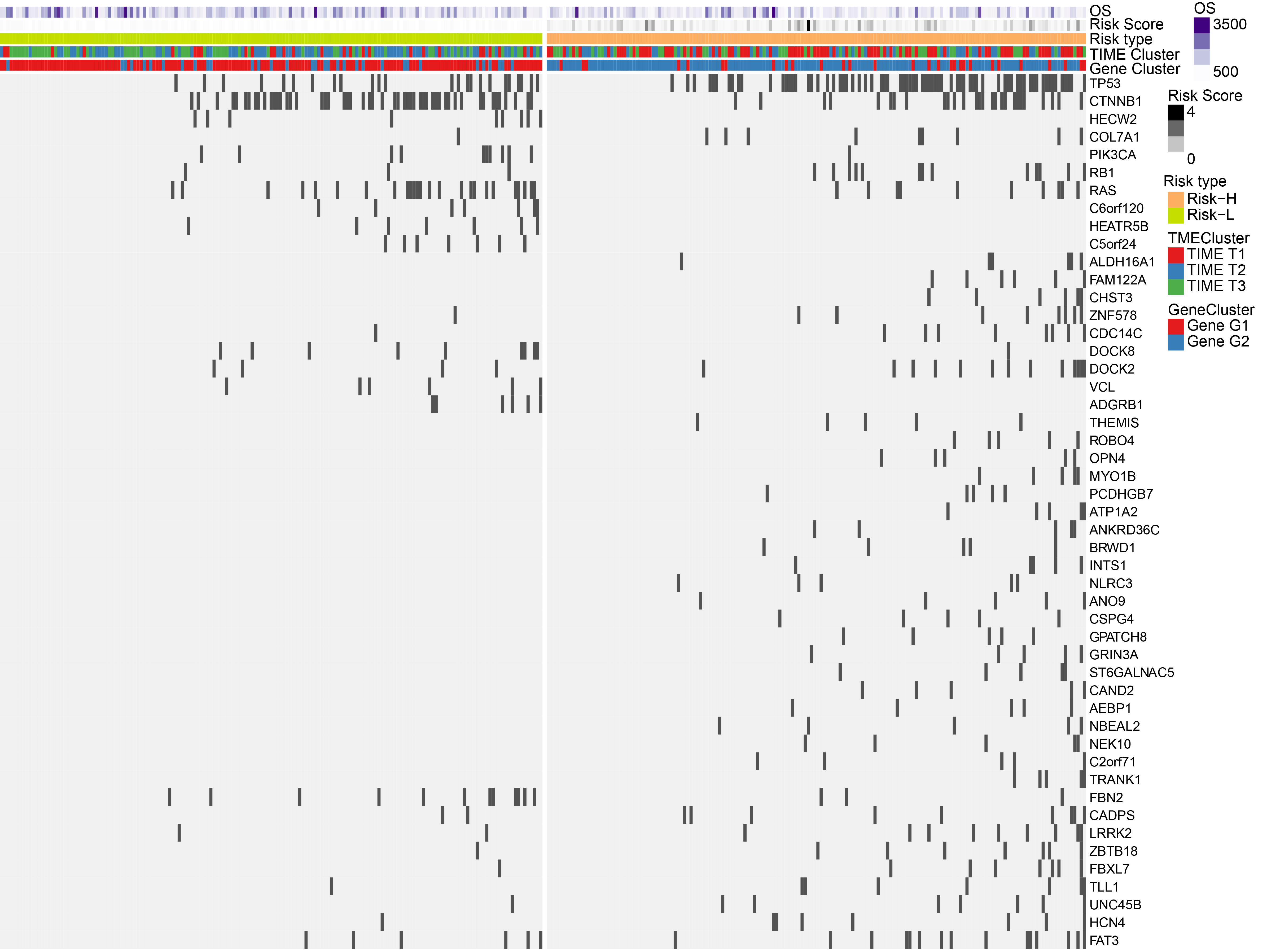

Supplement: Supplementary Figure 11 — Association between TS and somatic mutations. [file Image_11.tif]
